# 2,4,8,10,13-Penta­methyl-6-phenyl-13,14-dihydro-12*H*-6λ^5^-dibenzo[*d*,*i*][1,3,7,2]dioxaza­phosphecin-6-thione

**DOI:** 10.1107/S1600536809052180

**Published:** 2009-12-12

**Authors:** M. Krishnaiah, V.H.H. Surendra Babu, A. Uma Ravi Sankar, C. Naga Raju, Rajni Kant

**Affiliations:** a Department of Physics, S.V. University, Tirupati 517 502, India; b Japan Association for the Advancement of Medical Equipment, Hongo Bunkyo-ku, Tokyo-113 0033, Japan; c Department of Chemistry, S.V. University, Tirupati 517 502, India; d Department of Physics, University of Jammu, Jammu Tawi 180 006, India

## Abstract

In the title compound, C_25_H_28_NO_2_PS, the cyclo­decene ring exhibits a crown conformation. The two dimethyl­benzene rings which are fused symmetrically on either side of the ten-membered ring, make dihedral angles of 20.2 (1) and 18.0 (1)°. The phenyl ring substituted at P is perpendicular to the heterocyclic ring, making a dihedral angle of 88.4 (1)°. The crystal structure is stabilized by very weak intra­molecular C—H⋯O hydrogen bonding.

## Related literature

For applications of phospho­rus containing macrocycles, see: Lehn (1988 ▶); Cram (1988 ▶). For their biological activity, see: Sankar *et al.* (2009 ▶). For P=S bond lengths in related structures, see: Dutasta *et al.* (1979 ▶).
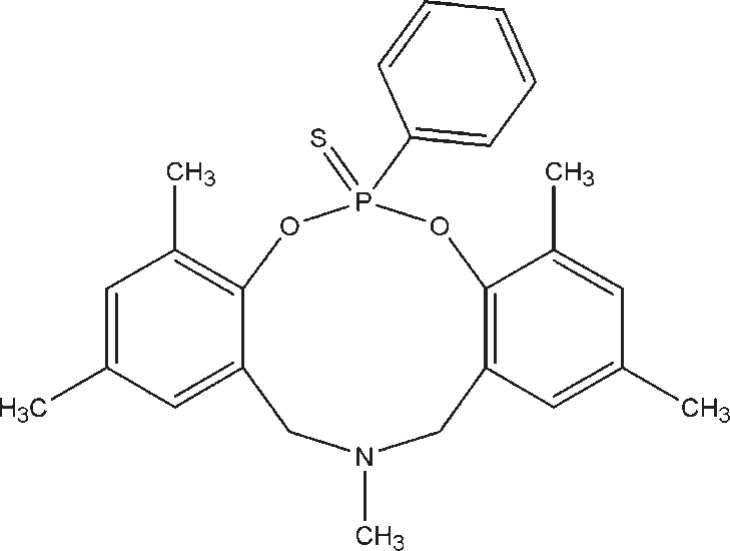


## Experimental

### 

#### Crystal data

C_25_H_28_NO_2_PS
*M* *_r_* = 437.52
Monoclinic, 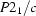

*a* = 8.7117 (9) Å
*b* = 16.3225 (16) Å
*c* = 16.9021 (16) Å
β = 99.525 (10)°
*V* = 2370.3 (4) Å^3^
*Z* = 4
Mo *K*α radiation
μ = 0.23 mm^−1^
*T* = 293 K
0.30 × 0.24 × 0.18 mm

#### Data collection

Oxford Diffraction Xcalibur diffractometer
21854 measured reflections
7063 independent reflections
3672 reflections with *I* > 2σ(*I*)
*R*_int_ = 0.033

#### Refinement

*R*[*F* ^2^ > 2σ(*F* ^2^)] = 0.049
*wR*(*F* ^2^) = 0.161
*S* = 1.03
7063 reflections
271 parameters
H-atom parameters constrained
Δρ_max_ = 0.35 e Å^−3^
Δρ_min_ = −0.29 e Å^−3^

### 

Data collection: *CryAlis PRO* (Oxford Diffraction, 2007 ▶); cell refinement: *CryAlis PRO*; data reduction: *CryAlis RED* (Oxford Diffraction, 2007 ▶); program(s) used to solve structure: *SHELXS86* (Sheldrick, 2008 ▶); program(s) used to refine structure: *SHELXL97* (Sheldrick, 2008 ▶); molecular graphics: *ZORTEPII* (Zsolnai, 1997 ▶); software used to prepare material for publication: *PARST* (Nardelli, 1995 ▶).

## Supplementary Material

Crystal structure: contains datablocks global, I. DOI: 10.1107/S1600536809052180/pb2015sup1.cif

Structure factors: contains datablocks I. DOI: 10.1107/S1600536809052180/pb2015Isup2.hkl

Additional supplementary materials:  crystallographic information; 3D view; checkCIF report

## Figures and Tables

**Table 1:** Hydrogen-bond geometry (Å, °)

*D*—H⋯*A*	*D*—H	H⋯*A*	*D*⋯*A*	*D*—H⋯*A*
C23—H23*A*⋯O3	0.96	2.39	2.848 (3)	109

**Fig. 1.**
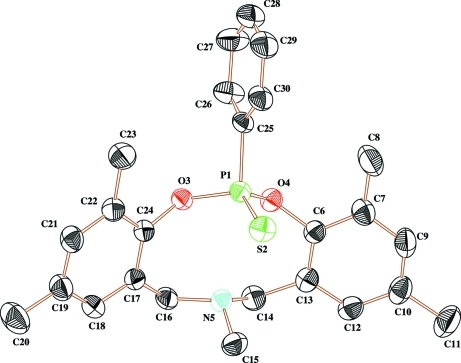
View of the molecule showing the atom-labelling scheme. Displacement ellipsoids are drawn at the 40% probability level.

**Fig. 2.**
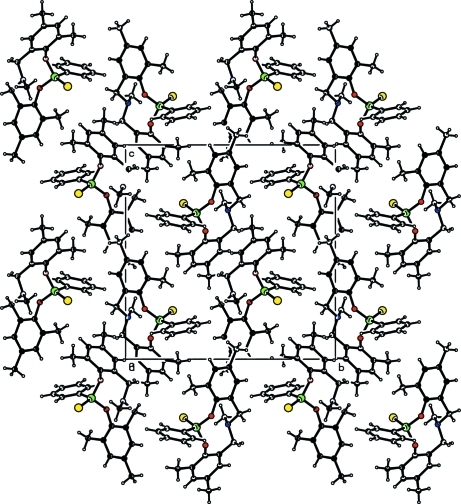
Packing view of the molecules in the unit cell.

## supplementary crystallographic information

### Comment

Phosphorus containing macrocycles are interesting molecules with potential applications in supramolecular and synthetic organic chemistry. The molecules found numerous industrial (Lehn, 1988) and biological (Cram, 1988) applications and also function as good hosts in the host–guest chemistry. The present title compound possesses antimicrobial activity against gram positive *Staphylococcus aureus*, gram negative *Escherichia coli* and antifungal activity against *Aspergillus niger*, *Helminthosporium oryzae*. It also exhibits equal antimicrobial and antifungal activities when compared with that of standard Penicillin and Griseofulvin (Sankar *et al.*, 2009).

The P=S bond length, 1.913Å is in good agreement with the related structure (Dutasta *et al.*, 1979). The crown conformation makes P—N bond length[3.133 Å] smaller than the Van der Waal's radii[3.35 Å] in such a way, which is favourable for P—N coordination. It is interesting to mention that the geometrical parameters between the two fragments from P to N of the ten membered heterocyclic ring are equal within the experimental limitations. The sulfur substituted at P and the methyl substituted at N are almost orthogonally oriented to the mean plane of cyclodecene. The bond angles O(3)—P(1)=S(2) and O(4)—P(1)=S(2) are identical to each other and same is the case with the bond angles O(3)—P(1)—C(25), O(4)—P(1)—C(25)and C(14)—N(5)—C(15),*C*(16)—N(5)—C(15). The crystal structure is stabilized by intra molecular C—H···O hydrogen bonding. The packing of the molecules is along [110] plane (figure 2).

### Experimental

A solution of phenyl dichlorophosphine (300 mg, 2 mmol) in 25 ml of dry toluene was added dropwise over a period of 20 minutes to a stirred solution of bis(2,4-dimethyl-2-hydroxybenzyl)methylamine (600 mg, 2 mmol) and triethyl amine (404 mg, 4 mmol) in 25 ml of dry toluene at 0°C under N_2_ atmosphere. After the addition, the temperature of the reaction mixture was raised to room temperature and stirred for 3 h, later the reaction mixture was stirred at 30°C for another 3 h. The triethylamine hydrochloride was removed by filtration. The intermediate obtained was dissolved in dry toluene (30 ml) and sulfur was added at room temperature. The reaction mixture was brought to reflux and kept with stirring for 2 h for the completion of reaction was indicated by TLC analysis. The solvent was removed in a rota-evaporator. The resulting crude product was crystallized by 2-propanol, rectangular shaped single crystals are obtained for diffraction studies.

### Refinement

All the H-atoms bound to carbon were positioned geometrically and refined using a riding model with d(C—H) = 0.93 Å, *U*_iso_=1.2_eq_ (C) for aromatic, 0.97 Å, *U*_iso_ = 1.2_eq_ (C)for CH_2_ group and 0.96 Å, *U*_iso_ = 1.5_eq_ (C) for CH_3_ group

### Crystal data

**Table:**

C_25_H_28_NO_2_PS	*F*(000) = 928
*M**_r_* = 437.52	*D*_x_ = 1.226 Mg m^−^^3^*D*_m_ = 1.225 Mg m^−^^3^*D*_m_ measured by not measured
Monoclinic, *P*2_1_/*c*	Mo *K*α radiation, λ = 0.71073 Å
Hall symbol: -P 2ybc	Cell parameters from 7063 reflections
*a* = 8.7117 (9) Å	θ = 3.1–30.2°
*b* = 16.3225 (16) Å	µ = 0.23 mm^−^^1^
*c* = 16.9021 (16) Å	*T* = 293 K
β = 99.525 (10)°	Rectangular, brown
*V* = 2370.3 (4) Å^3^	0.30 × 0.24 × 0.18 mm
*Z* = 4	

### Data collection

**Table:**

Oxford Diffraction Xcalibur diffractometer	3672 reflections with *I* > 2σ(*I*)
Radiation source: fine-focus sealed tube	*R*_int_ = 0.033
graphite	θ_max_ = 30.2°, θ_min_ = 3.1°
ω–2θ scans	*h* = −8→12
21854 measured reflections	*k* = −23→20
7063 independent reflections	*l* = −23→23

### Refinement

**Table:**

Refinement on *F*^2^	Primary atom site location: structure-invariant direct methods
Least-squares matrix: full	Secondary atom site location: difference Fourier map
*R*[*F*^2^ > 2σ(*F*^2^)] = 0.049	Hydrogen site location: inferred from neighbouring sites
*wR*(*F*^2^) = 0.161	H-atom parameters constrained
*S* = 1.03	*w* = 1/[σ^2^(*F*_o_^2^) + (0.0805*P*)^2^] where *P* = (*F*_o_^2^ + 2*F*_c_^2^)/3
7063 reflections	(Δ/σ)_max_ < 0.001
271 parameters	Δρ_max_ = 0.35 e Å^−^^3^
0 restraints	Δρ_min_ = −0.29 e Å^−^^3^

### Special details

**Table:**

Geometry. All e.s.d.'s (except the e.s.d. in the dihedral angle between two l.s. planes) are estimated using the full covariance matrix. The cell e.s.d.'s are taken into account individually in the estimation of e.s.d.'s in distances, angles and torsion angles; correlations between e.s.d.'s in cell parameters are only used when they are defined by crystal symmetry. An approximate (isotropic) treatment of cell e.s.d.'s is used for estimating e.s.d.'s involving l.s. planes.
Refinement. Refinement of *F*^2^ against ALL reflections. The weighted *R*-factor *wR* and goodness of fit *S* are based on *F*^2^, conventional *R*-factors *R* are based on *F*, with *F* set to zero for negative *F*^2^. The threshold expression of *F*^2^ > σ(*F*^2^) is used only for calculating *R*-factors(gt) *etc*. and is not relevant to the choice of reflections for refinement. *R*-factors based on *F*^2^ are statistically about twice as large as those based on *F*, and *R*- factors based on ALL data will be even larger.

### Fractional atomic coordinates and isotropic or equivalent isotropic displacement parameters (Å^2^)

**Table:**

	*x*	*y*	*z*	*U*_iso_*/*U*_eq_	
P1	0.17744 (6)	0.16344 (3)	0.18237 (3)	0.03883 (16)	
S2	0.02473 (7)	0.22402 (3)	0.22679 (4)	0.05506 (19)	
O3	0.12396 (15)	0.11944 (8)	0.09791 (7)	0.0405 (3)	
C25	0.3425 (2)	0.22061 (12)	0.16102 (11)	0.0427 (5)	
O4	0.26513 (16)	0.09104 (8)	0.23448 (8)	0.0439 (3)	
N5	−0.0375 (2)	0.01189 (10)	0.19579 (9)	0.0424 (4)	
C24	−0.0253 (2)	0.12021 (12)	0.05097 (11)	0.0410 (5)	
C22	−0.0613 (3)	0.18239 (12)	−0.00492 (12)	0.0470 (5)	
C13	0.1521 (3)	−0.00145 (13)	0.31893 (12)	0.0452 (5)	
C17	−0.1224 (2)	0.05350 (12)	0.05584 (11)	0.0426 (5)	
C6	0.2449 (2)	0.06607 (13)	0.31197 (11)	0.0427 (5)	
C16	−0.0717 (3)	−0.01544 (12)	0.11289 (11)	0.0466 (5)	
H16A	−0.1533	−0.0565	0.1078	0.056*	
H16B	0.0204	−0.0409	0.0985	0.056*	
C18	−0.2601 (3)	0.05058 (14)	0.00225 (12)	0.0524 (5)	
H18	−0.3272	0.0067	0.0048	0.063*	
C14	0.0690 (3)	−0.04331 (13)	0.24529 (12)	0.0512 (5)	
H14A	0.1447	−0.0640	0.2142	0.061*	
H14B	0.0111	−0.0897	0.2610	0.061*	
C21	−0.2018 (3)	0.17554 (14)	−0.05738 (13)	0.0550 (6)	
H21	−0.2290	0.2164	−0.0955	0.066*	
C30	0.3574 (3)	0.30281 (13)	0.17657 (13)	0.0544 (6)	
H30	0.2791	0.3311	0.1963	0.065*	
C12	0.1470 (3)	−0.03023 (14)	0.39559 (13)	0.0525 (6)	
H12	0.0850	−0.0754	0.4017	0.063*	
C29	0.4898 (3)	0.34342 (16)	0.16272 (16)	0.0694 (7)	
H29	0.4999	0.3992	0.1734	0.083*	
C23	0.0493 (3)	0.25157 (14)	−0.01172 (15)	0.0670 (7)	
H23A	0.1391	0.2463	0.0294	0.100*	
H23B	−0.0015	0.3028	−0.0054	0.100*	
H23C	0.0810	0.2499	−0.0635	0.100*	
C7	0.3294 (3)	0.10556 (14)	0.37772 (13)	0.0561 (6)	
C15	−0.1759 (3)	0.02847 (16)	0.22984 (13)	0.0607 (6)	
H15A	−0.1467	0.0470	0.2842	0.091*	
H15B	−0.2367	−0.0207	0.2291	0.091*	
H15C	−0.2363	0.0701	0.1989	0.091*	
C19	−0.3022 (3)	0.11051 (15)	−0.05507 (13)	0.0575 (6)	
C26	0.4609 (3)	0.17977 (15)	0.13089 (15)	0.0617 (6)	
H26	0.4525	0.1239	0.1200	0.074*	
C10	0.2310 (3)	0.00598 (16)	0.46344 (13)	0.0598 (6)	
C20	−0.4511 (3)	0.1035 (2)	−0.11439 (17)	0.0885 (9)	
H20A	−0.5047	0.0543	−0.1040	0.133*	
H20B	−0.4274	0.1016	−0.1678	0.133*	
H20C	−0.5159	0.1501	−0.1092	0.133*	
C9	0.3187 (3)	0.07330 (17)	0.45288 (13)	0.0641 (7)	
H9	0.3739	0.0988	0.4980	0.077*	
C8	0.4268 (4)	0.17912 (18)	0.36884 (17)	0.0832 (9)	
H8A	0.4759	0.1975	0.4209	0.125*	
H8B	0.3622	0.2220	0.3426	0.125*	
H8C	0.5051	0.1652	0.3373	0.125*	
C28	0.6049 (4)	0.30329 (19)	0.13385 (16)	0.0750 (8)	
H28	0.6938	0.3313	0.1255	0.090*	
C27	0.5903 (3)	0.22253 (18)	0.11724 (18)	0.0758 (8)	
H27	0.6685	0.1954	0.0963	0.091*	
C11	0.2268 (4)	−0.0284 (2)	0.54643 (14)	0.0864 (9)	
H11A	0.1601	−0.0756	0.5421	0.130*	
H11B	0.1877	0.0125	0.5787	0.130*	
H11C	0.3301	−0.0440	0.5710	0.130*	

### Atomic displacement parameters (Å^2^)

**Table:**

	*U*^11^	*U*^22^	*U*^33^	*U*^12^	*U*^13^	*U*^23^
P1	0.0388 (3)	0.0353 (3)	0.0422 (3)	−0.0001 (2)	0.0060 (2)	−0.0017 (2)
S2	0.0534 (4)	0.0493 (3)	0.0648 (4)	0.0089 (3)	0.0168 (3)	−0.0070 (3)
O3	0.0393 (8)	0.0403 (8)	0.0416 (7)	−0.0017 (6)	0.0058 (6)	−0.0024 (6)
C25	0.0435 (12)	0.0398 (11)	0.0432 (10)	−0.0097 (9)	0.0021 (9)	−0.0006 (9)
O4	0.0452 (8)	0.0433 (8)	0.0429 (7)	0.0045 (6)	0.0060 (6)	0.0041 (6)
N5	0.0452 (10)	0.0415 (9)	0.0400 (9)	0.0009 (8)	0.0056 (8)	0.0041 (7)
C24	0.0408 (11)	0.0446 (11)	0.0373 (10)	−0.0019 (9)	0.0053 (9)	−0.0022 (9)
C22	0.0514 (13)	0.0424 (11)	0.0467 (11)	−0.0007 (10)	0.0066 (10)	0.0011 (9)
C13	0.0475 (12)	0.0426 (11)	0.0448 (11)	0.0105 (9)	0.0059 (10)	0.0064 (9)
C17	0.0472 (12)	0.0425 (11)	0.0381 (10)	−0.0061 (9)	0.0075 (9)	−0.0018 (9)
C6	0.0408 (11)	0.0456 (11)	0.0413 (10)	0.0051 (9)	0.0052 (9)	0.0046 (9)
C16	0.0541 (13)	0.0377 (11)	0.0466 (11)	−0.0121 (10)	0.0039 (10)	−0.0019 (9)
C18	0.0485 (13)	0.0562 (13)	0.0507 (12)	−0.0104 (11)	0.0030 (11)	−0.0025 (10)
C14	0.0596 (14)	0.0405 (12)	0.0518 (12)	−0.0019 (10)	0.0039 (11)	0.0062 (10)
C21	0.0571 (15)	0.0555 (14)	0.0485 (12)	0.0048 (11)	−0.0028 (11)	0.0085 (10)
C30	0.0634 (15)	0.0398 (12)	0.0576 (13)	−0.0106 (11)	0.0032 (11)	−0.0025 (10)
C12	0.0471 (13)	0.0586 (14)	0.0529 (13)	0.0067 (10)	0.0114 (11)	0.0123 (11)
C29	0.085 (2)	0.0476 (14)	0.0724 (16)	−0.0216 (14)	0.0036 (15)	0.0030 (12)
C23	0.0761 (18)	0.0540 (14)	0.0669 (15)	−0.0114 (13)	0.0004 (14)	0.0196 (12)
C7	0.0539 (14)	0.0583 (14)	0.0525 (12)	0.0011 (11)	−0.0021 (11)	0.0016 (11)
C15	0.0491 (14)	0.0803 (17)	0.0547 (13)	−0.0015 (12)	0.0143 (11)	0.0081 (12)
C19	0.0548 (14)	0.0613 (15)	0.0528 (13)	−0.0014 (12)	−0.0019 (11)	0.0010 (11)
C26	0.0509 (14)	0.0508 (14)	0.0871 (17)	−0.0071 (11)	0.0224 (13)	−0.0073 (12)
C10	0.0577 (15)	0.0775 (17)	0.0440 (12)	0.0175 (13)	0.0075 (11)	0.0116 (12)
C20	0.0703 (19)	0.094 (2)	0.088 (2)	−0.0080 (16)	−0.0243 (16)	0.0151 (17)
C9	0.0643 (16)	0.0811 (18)	0.0430 (12)	0.0054 (14)	−0.0025 (11)	−0.0043 (12)
C8	0.089 (2)	0.0820 (19)	0.0676 (16)	−0.0263 (16)	−0.0194 (15)	0.0023 (15)
C28	0.0702 (19)	0.081 (2)	0.0722 (17)	−0.0307 (16)	0.0064 (15)	0.0094 (15)
C27	0.0569 (17)	0.080 (2)	0.096 (2)	−0.0119 (14)	0.0299 (15)	−0.0035 (16)
C11	0.079 (2)	0.130 (3)	0.0516 (14)	0.0177 (19)	0.0149 (14)	0.0249 (16)

### Geometric parameters (Å, °)

**Table:**

P1—O4	1.5916 (14)	C12—C10	1.387 (3)
P1—O3	1.5970 (13)	C12—H12	0.9300
P1—C25	1.800 (2)	C29—C28	1.355 (4)
P1—S2	1.9083 (7)	C29—H29	0.9300
O3—C24	1.407 (2)	C23—H23A	0.9600
C25—C30	1.369 (3)	C23—H23B	0.9600
C25—C26	1.393 (3)	C23—H23C	0.9600
O4—C6	1.410 (2)	C7—C9	1.393 (3)
N5—C15	1.445 (3)	C7—C8	1.492 (4)
N5—C16	1.454 (2)	C15—H15A	0.9600
N5—C14	1.454 (3)	C15—H15B	0.9600
C24—C22	1.387 (3)	C15—H15C	0.9600
C24—C17	1.390 (3)	C19—C20	1.507 (3)
C22—C21	1.391 (3)	C26—C27	1.378 (3)
C22—C23	1.501 (3)	C26—H26	0.9300
C13—C6	1.383 (3)	C10—C9	1.367 (4)
C13—C12	1.386 (3)	C10—C11	1.517 (3)
C13—C14	1.498 (3)	C20—H20A	0.9600
C17—C18	1.379 (3)	C20—H20B	0.9600
C17—C16	1.500 (3)	C20—H20C	0.9600
C6—C7	1.386 (3)	C9—H9	0.9300
C16—H16A	0.9700	C8—H8A	0.9600
C16—H16B	0.9700	C8—H8B	0.9600
C18—C19	1.383 (3)	C8—H8C	0.9600
C18—H18	0.9300	C28—C27	1.349 (4)
C14—H14A	0.9700	C28—H28	0.9300
C14—H14B	0.9700	C27—H27	0.9300
C21—C19	1.380 (3)	C11—H11A	0.9600
C21—H21	0.9300	C11—H11B	0.9600
C30—C29	1.383 (3)	C11—H11C	0.9600
C30—H30	0.9300		
			
O4—P1—O3	101.72 (7)	C28—C29—H29	119.4
O4—P1—C25	99.77 (9)	C30—C29—H29	119.4
O3—P1—C25	100.28 (8)	C22—C23—H23A	109.5
O4—P1—S2	117.97 (6)	C22—C23—H23B	109.5
O3—P1—S2	117.74 (6)	H23A—C23—H23B	109.5
C25—P1—S2	116.25 (7)	C22—C23—H23C	109.5
C24—O3—P1	127.29 (12)	H23A—C23—H23C	109.5
C30—C25—C26	119.1 (2)	H23B—C23—H23C	109.5
C30—C25—P1	121.54 (17)	C6—C7—C9	116.7 (2)
C26—C25—P1	119.30 (16)	C6—C7—C8	121.9 (2)
C6—O4—P1	127.32 (12)	C9—C7—C8	121.4 (2)
C15—N5—C16	112.94 (17)	N5—C15—H15A	109.5
C15—N5—C14	112.43 (16)	N5—C15—H15B	109.5
C16—N5—C14	111.96 (16)	H15A—C15—H15B	109.5
C22—C24—C17	122.84 (19)	N5—C15—H15C	109.5
C22—C24—O3	118.35 (17)	H15A—C15—H15C	109.5
C17—C24—O3	118.33 (17)	H15B—C15—H15C	109.5
C24—C22—C21	116.83 (19)	C21—C19—C18	117.9 (2)
C24—C22—C23	121.8 (2)	C21—C19—C20	121.3 (2)
C21—C22—C23	121.30 (19)	C18—C19—C20	120.8 (2)
C6—C13—C12	117.3 (2)	C27—C26—C25	119.7 (2)
C6—C13—C14	120.14 (18)	C27—C26—H26	120.2
C12—C13—C14	122.4 (2)	C25—C26—H26	120.2
C18—C17—C24	117.39 (19)	C9—C10—C12	117.7 (2)
C18—C17—C16	121.85 (18)	C9—C10—C11	121.2 (2)
C24—C17—C16	120.57 (18)	C12—C10—C11	121.1 (2)
C13—C6—C7	122.87 (19)	C19—C20—H20A	109.5
C13—C6—O4	118.29 (18)	C19—C20—H20B	109.5
C7—C6—O4	118.58 (19)	H20A—C20—H20B	109.5
N5—C16—C17	112.40 (16)	C19—C20—H20C	109.5
N5—C16—H16A	109.1	H20A—C20—H20C	109.5
C17—C16—H16A	109.1	H20B—C20—H20C	109.5
N5—C16—H16B	109.1	C10—C9—C7	123.1 (2)
C17—C16—H16B	109.1	C10—C9—H9	118.4
H16A—C16—H16B	107.9	C7—C9—H9	118.4
C17—C18—C19	122.4 (2)	C7—C8—H8A	109.5
C17—C18—H18	118.8	C7—C8—H8B	109.5
C19—C18—H18	118.8	H8A—C8—H8B	109.5
N5—C14—C13	111.73 (17)	C7—C8—H8C	109.5
N5—C14—H14A	109.3	H8A—C8—H8C	109.5
C13—C14—H14A	109.3	H8B—C8—H8C	109.5
N5—C14—H14B	109.3	C27—C28—C29	119.8 (3)
C13—C14—H14B	109.3	C27—C28—H28	120.1
H14A—C14—H14B	107.9	C29—C28—H28	120.1
C19—C21—C22	122.6 (2)	C28—C27—C26	120.8 (3)
C19—C21—H21	118.7	C28—C27—H27	119.6
C22—C21—H21	118.7	C26—C27—H27	119.6
C25—C30—C29	119.5 (2)	C10—C11—H11A	109.5
C25—C30—H30	120.2	C10—C11—H11B	109.5
C29—C30—H30	120.2	H11A—C11—H11B	109.5
C13—C12—C10	122.3 (2)	C10—C11—H11C	109.5
C13—C12—H12	118.8	H11A—C11—H11C	109.5
C10—C12—H12	118.8	H11B—C11—H11C	109.5
C28—C29—C30	121.1 (2)		
			
O4—P1—O3—C24	130.89 (15)	C24—C17—C18—C19	0.5 (3)
C25—P1—O3—C24	−126.77 (16)	C16—C17—C18—C19	−174.6 (2)
S2—P1—O3—C24	0.33 (17)	C15—N5—C14—C13	−74.0 (2)
O4—P1—C25—C30	−127.47 (18)	C16—N5—C14—C13	157.61 (17)
O3—P1—C25—C30	128.60 (18)	C6—C13—C14—N5	−59.9 (3)
S2—P1—C25—C30	0.5 (2)	C12—C13—C14—N5	123.5 (2)
O4—P1—C25—C26	50.00 (19)	C24—C22—C21—C19	0.0 (3)
O3—P1—C25—C26	−53.93 (19)	C23—C22—C21—C19	176.8 (2)
S2—P1—C25—C26	177.98 (16)	C26—C25—C30—C29	−0.5 (3)
O3—P1—O4—C6	−131.31 (16)	P1—C25—C30—C29	176.95 (18)
C25—P1—O4—C6	125.93 (17)	C6—C13—C12—C10	−0.3 (3)
S2—P1—O4—C6	−0.90 (18)	C14—C13—C12—C10	176.4 (2)
P1—O3—C24—C22	89.3 (2)	C25—C30—C29—C28	0.1 (4)
P1—O3—C24—C17	−98.4 (2)	C13—C6—C7—C9	0.8 (3)
C17—C24—C22—C21	0.0 (3)	O4—C6—C7—C9	−173.3 (2)
O3—C24—C22—C21	171.87 (18)	C13—C6—C7—C8	−179.0 (2)
C17—C24—C22—C23	−176.8 (2)	O4—C6—C7—C8	6.9 (3)
O3—C24—C22—C23	−4.9 (3)	C22—C21—C19—C18	0.3 (4)
C22—C24—C17—C18	−0.2 (3)	C22—C21—C19—C20	−178.1 (2)
O3—C24—C17—C18	−172.11 (17)	C17—C18—C19—C21	−0.6 (3)
C22—C24—C17—C16	174.92 (18)	C17—C18—C19—C20	177.8 (2)
O3—C24—C17—C16	3.0 (3)	C30—C25—C26—C27	0.0 (4)
C12—C13—C6—C7	−0.8 (3)	P1—C25—C26—C27	−177.6 (2)
C14—C13—C6—C7	−177.5 (2)	C13—C12—C10—C9	1.3 (3)
C12—C13—C6—O4	173.30 (18)	C13—C12—C10—C11	−178.2 (2)
C14—C13—C6—O4	−3.4 (3)	C12—C10—C9—C7	−1.3 (4)
P1—O4—C6—C13	99.7 (2)	C11—C10—C9—C7	178.1 (2)
P1—O4—C6—C7	−86.0 (2)	C6—C7—C9—C10	0.3 (4)
C15—N5—C16—C17	75.8 (2)	C8—C7—C9—C10	−179.9 (3)
C14—N5—C16—C17	−156.10 (18)	C30—C29—C28—C27	0.8 (4)
C18—C17—C16—N5	−125.8 (2)	C29—C28—C27—C26	−1.4 (4)
C24—C17—C16—N5	59.3 (3)	C25—C26—C27—C28	1.0 (4)

### Hydrogen-bond geometry (Å, °)

**Table:**

*D*—H···*A*	*D*—H	H···*A*	*D*···*A*	*D*—H···*A*
C23—H23A···O3	0.96	2.39	2.848 (3)	109
